# *Sox10-*Venus mice: a new tool for real-time labeling of neural crest lineage cells and oligodendrocytes

**DOI:** 10.1186/1756-6606-3-31

**Published:** 2010-10-31

**Authors:** Shinsuke Shibata, Akimasa Yasuda, Francois Renault-Mihara, Satoshi Suyama, Hiroyuki Katoh, Takayoshi Inoue, Yukiko U Inoue, Narihito Nagoshi, Momoka Sato, Masaya Nakamura, Chihiro Akazawa, Hideyuki Okano

**Affiliations:** 1 Department of Physiology, Keio University School of Medicine, Shinjuku-ku, Tokyo 160-8582, Japan; 2 Department of Orthopaedic Surgery, Keio University School of Medicine, Shinjuku-ku, Tokyo 160-8582, Japan; 3 Clinical Research Center, National Hospital Organization, Murayama Medical Center, 2-37-1 Gakuen, Musashimurayama, Tokyo 208-0011, Japan; 4 Department of Biochemistry and Cellular Biology, National Institute of Neuroscience, NCNP, Tokyo 187-8502, Japan; 5 Department of Biochemistry and Biophysics, Graduate School of Health and Sciences, Tokyo Medical and Dental University, Bunkyo, Tokyo, Japan

## Abstract

**Background:**

While several mouse strains have recently been developed for tracing neural crest or oligodendrocyte lineages, each strain has inherent limitations. The connection between human *SOX10 *mutations and neural crest cell pathogenesis led us to focus on the *Sox10 *gene, which is critical for neural crest development. We generated *Sox10-*Venus BAC transgenic mice to monitor Sox10 expression in both normal development and in pathological processes.

**Results:**

Tissue fluorescence distinguished neural crest progeny cells and oligodendrocytes in the *Sox10-*Venus mouse embryo. Immunohistochemical analysis confirmed that Venus expression was restricted to cells expressing endogenous Sox10. Time-lapse imaging of various tissues in *Sox10-*Venus mice demonstrated that Venus expression could be visualized at the single-cell level *in vivo *due to the intense, focused Venus fluorescence. In the adult *Sox10-*Venus mouse, several types of mature and immature oligodendrocytes along with Schwann cells were clearly labeled with Venus, both before and after spinal cord injury.

**Conclusions:**

In the newly-developed *Sox10-*Venus transgenic mouse, Venus fluorescence faithfully mirrors endogenous Sox10 expression and allows for *in vivo *imaging of live cells at the single-cell level. This *Sox10-*Venus mouse will thus be a useful tool for studying neural crest cells or oligodendrocytes, both in development and in pathological processes.

## Background

The neural crest (NC) is a transient embryonic tissue. NC cells delaminate from the dorsal neural tube as it closes [1] and migrate to distinct locations, where they differentiate into various cell types, including neurons, glia, melanocytes, endocrine cells, and mesenchymal cells [2-5].

The Sox proteins belong to the HMG (high mobility group) domain of transcription factors [6,7]. Sox-E is the earliest marker of a subset of cells at the border of the neural plate that will give rise to NC-lineage cells [8]. Sox10, which is a member of the Sox-E family and shares high sequence homology with other Sox-E member transcription factors, regulates and coordinates diverse developmental processes such as organ development and cell survival and specification. Sox10 is highly expressed in the emerging NC and later in the developing glial cells of the peripheral nervous system (PNS) and central nervous system (CNS) [9,10]. Whether in mice or humans, Sox family protein deletions or mutations often result in developmental defects and congenital disease, and mutations of the human *SOX10 *gene are associated with NC cell abnormalities [9,11-13].

Several transgenic mouse strains dedicated to tracing the NC lineage have already been developed, such as *Wnt1*-Cre [14], *Protein zero (P0)*-Cre [15], and *Ht-PA-*Cre [16] mice crossed with Cre-dependent reporter mice. The Cre recombinase expression was previously visualized by *LacZ*, a β-galactosidase reporter gene inserted in the ROSA26 locus, that is expressed only after the loxP-flanked intervening sequence is excised by Cre [17]. Once a specific promoter is activated, the cell is indelibly tagged with β-galactosidase. This kind of transgenic mouse is useful for monitoring the transient activation of various promoters, including the NC-specific promoter. Recently, mouse strains expressing a fluorescence-based reporter upon Cre-mediated conditional gene deletion have been developed for prospective cell sorting or direct observation without fixation [18]; the CAG-CAT-EGFP reporter transgenic mouse strain expresses enhanced green fluorescent protein (EGFP) when the loxP-flanked CAT gene located between the modified chicken β-actin promoter (CAG promoter) and the EGFP gene [18] is excised with Cre. In previous studies, we have used mice that enable Cre/loxP-mediated cell labeling with *LacZ *or EGFP to analyze the NC lineage and to trace NC cells after their migration and differentiation [5,19-21].

However, for specific gene regulation analysis, transgenic or knock-in mouse lines that express a specific gene profile *in vivo *are more useful, because the reporter gene is expressed only while the specific promoter or enhancer is active, and ceases when the promoter becomes inactive. Reporter mice have recently been developed to evaluate cell-type specification and maturation in the oligodendroglial lineage; these are the 2'-3'-cyclic nucleotide 3'-phosphodiesterase (*CNP*)-EGFP and myelin proteolipid protein (*PLP*)-EGFP transgenic mouse lines [22,23]. The *CNP*-EGFP transgenic mouse, in which the CNP promoter controls EGFP expression, has been used for the prospective identification of live oligodendroglial cells both *in vivo *and *in vitro *[23]. The *PLP*-EGFP transgenic mouse, in which EGFP expression is driven by the mouse *PLP *gene promoter, has also been developed for investigating oligodendrocyte lineage cells without fixation and immunostaining [22].

Sox10 expression is closely related to NC-lineage cells. The *Sox10*^*LacZ*/+ ^[24], *Sox10-*rtTA [25], and *Sox10-*Cre [26] mouse lines have all been reported to label NC cells and oligodendrocytes. *Sox10*^*LacZ*/+^[24], a mutant mouse targeting *Sox10*, was generated by replacing the open reading frame of *Sox10 *with *lacZ *sequences. The *Sox10*^*LacZ*/+ ^mutation causes haploinsufficiency, in which even heterozygous pups have the phenotype found in mice with a spontaneous mutation in the *Sox10 *allele. Although *LacZ *expression in this knock-in strain faithfully reflects the endogenous Sox10 expression, it is difficult to observe normal developmental behavior in the labeled cells because of the abnormal and pathological condition of the *Sox10^LacZ/LacZ ^*homozygous mice [24]. Another unique reporter strain is the *Sox10-*rtTA knock-in mouse [25], in which a variant of the reverse tetracycline-controlled transactivator (rtTA) is inserted into the genomic *Sox10 *locus, and the mice are crossed with the doxycycline-dependent *LacZ *reporter line. This strain correctly recapitulates endogenous Sox10 expression in the NC and its derivatives, and also in oligodendrocytes. This inducible transgenic system is limited in its range of analysis, because the reporter gene expression is temporary and requires X-gal staining [25]. The *Sox10-*Cre transgenic mouse strain, designated as the S4F:Cre mouse, when crossed with the reporter line strain Rosa-*LacZ*, identifies cells expressing Sox10, including NC-derived cells, oligodendrocytes, and cells in the ventral neural tube [26]. These strains are powerful tools for tracing the progeny of Sox10-expressing cells in analyses of NC cell migration and oligodendroglial differentiation. However, permanent reporter gene expression does not permit the real-time analysis of Sox10 expression. To overcome these limitations, we generated a new *Sox10-*Venus transgenic mouse, and confirmed that it enables the normal behavior of Sox10-expressing cells to be observed *in vivo*.

## Results

### *Sox10-*Venus BAC transgenic mouse generation

To examine the Sox10 expression profile *in vivo*, we took advantage of the bacterial artificial chromosome (BAC) transgenic strategy where entire regulatory machinery for a given gene expression might be covered with a single BAC clone. In short, we modified a 225.6 kb-sized BAC clone *RP24-85O14 *by means of homologous recombination to harbor the coding sequence of the fluorescent Venus protein [27] in-frame to the *Sox10 *translation initiation codon (ATG) that locates at the middle portion of the clone. This construct yielded seven BAC transgenic founders expressing Venus and we hereafter analyzed the complete expression profile in a particular transgenic founder with the brightest illumination.

**Figure 1:**
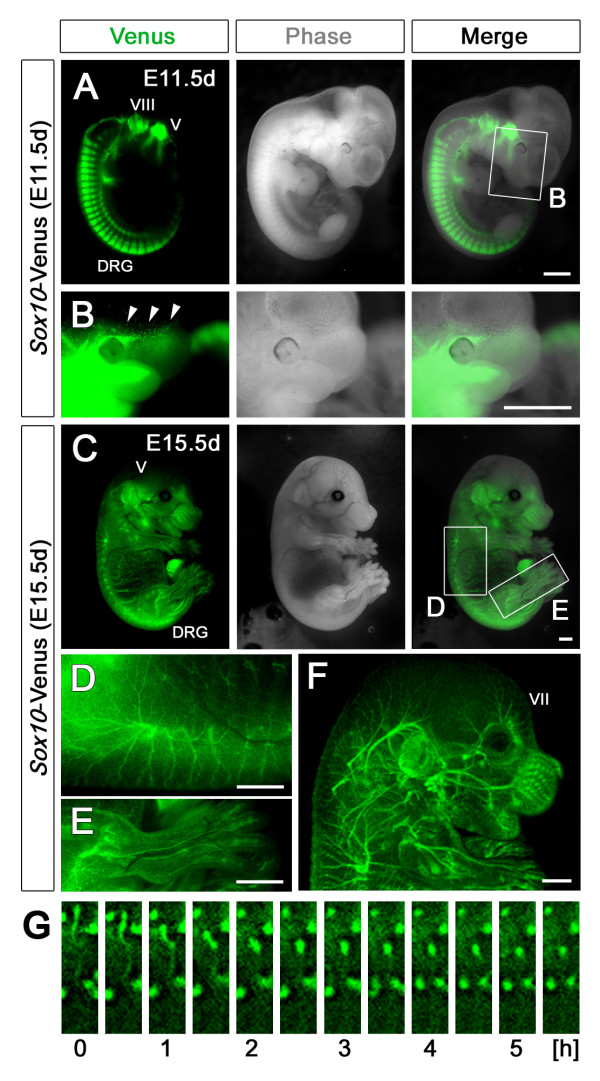
**The novel *Sox10-*Venus transgenic mouse strain**. The *Sox10-*Venus BAC transgenic mouse strain was generated to investigate the Sox10 expression profile *in vivo*. (A) Venus could be detected in NC lineage tissues, including the DRG and trigeminal ganglia, in E11.5 d mice. Sox10^+ ^cells that were not NC-derived, including cells in the otic vesicle, could also be visualized with Venus. (B) High-intensity fluorescence enabled us to observe a migration stream at the single-cell level, from outside the embryo. (C) At E15.5 d, deep-tissue Venus fluorescence decreased. At this age, migrating Venus^+ ^cells and the network formation of Venus^+ ^cells in the superficial skin layer could be traced. (D) The posterior branches of the spinal nerve were clearly visualized with Venus^+ ^Schwann cells, along with peripheral nerve fibers. (E) In the hind limb, the peripheral nerve and vascular network formations were clearly outlined. (F) The peripheral neural network mainly derived from cranial nerve VII could be visualized with three-dimensional reconstruction with an Olympus MVX-CSU microscope. (G) Time-lapse imaging of the front facial area of embryonic *Sox10-*Venus mice clearly showed individual Venus^+ ^cell movement (also shown in movies in Additional Files 2 and 3). (B) and (D, E) are high-magnification images of the indicated areas in Figures (A) and (C), respectively. Cranial nerves V, trigeminal nerve; VII, facial nerve; VIII, vestibulocochlear nerve, Scale bars; (A-F) 1.0 mm.

First, we evaluated the distribution of Venus fluorescence at each developmental stage of the *Sox10*-Venus mouse embryo. Venus fluorescence in classical NC lineage tissues was directly visible from outside the embryo by using an epifluorescence microscope and a UV light. At embryonic day 11.5 (E11.5 d), Venus green fluorescence was particularly intense in the dorsal root ganglia (DRG) and the trigeminal ganglia (cranial nerve V) (Figure 1A). We observed that Venus fluorescence was not limited to classical NC lineage tissue, but was also observed in non-NC tissues, including the otic vesicle. This is consistent with previous studies in which *in situ *hybridization revealed endogenous Sox10 expression in otic vesicle cells [28,29], which are the primitive state of the vestibulocochlear nucleus (cranial nerve VIII). In the facial area of the E11.5 d embryo, aggregated green cells were observed among scattered Venus-positive cells (Figure 1B). The intensity and resolution of the Venus fluorescence allowed us to observe single cells in a pattern suggestive of the NC cell rostral migration process (arrowheads in Figure 1B). As development proceeded from E11.5 d to E15.5 d, Venus fluorescence from the DRGs and nuclei of the cranial nerves located in deep tissues gradually decreased, due to the thickening dermal layer (Figure 1A, C, and Additional file 1A - 1C). However, migrating Venus^+ ^cells in the superficial layer of the embryonic skin were now detectable from outside the body. Venus fluorescence was observed in the Schwann cells of the neural network stemming from the spinal nerve's posterior branches, and in peripheral sensory nerve fibers (Figure 1D). In the hind limb, the peripheral nerve network and the adjacent vascular network were visible (Figure 1E). Three-dimensional reconstructions of the peripheral nerve fibers displayed the entire neural network in the craniofacial area, which was mainly derived from the branches of the facial nerve (cranial nerve VII) (Figure 1F).

### Observing the behavior of individual *Sox10-*Venus cells

The intensity and resolution of the Venus fluorescence in the new *Sox10-*Venus mice led us to evaluate whether it was possible to monitor the behavior of individual cells. Time-lapse imaging *ex-vivo *in skin explants from E14.5 d *Sox10-*Venus embryos was first performed using an epifluorescence microscope. Within this experimental setup, it was easy to detect and follow the movements of single cells (Figure 1G and the movie in Additional File 2). As Sox10 is expressed in deep tissues during the early stages of development, we next examined whether it would be possible to observe Venus fluorescence in whole *Sox10-*Venus embryos. Whole embryos at E10.5 d were observed using a confocal microscope, and followed over time. Time-lapse imaging of the front facial area allowed us to monitor the migration of several Venus^+ ^cells within the embryo over a duration of several hours. We were also able to observe the shape dynamics of individual migrating cells (movie in Additional File 3).

### Venus fluorescence in frozen sections of *Sox10-*Venus mice

**Figure 2:**
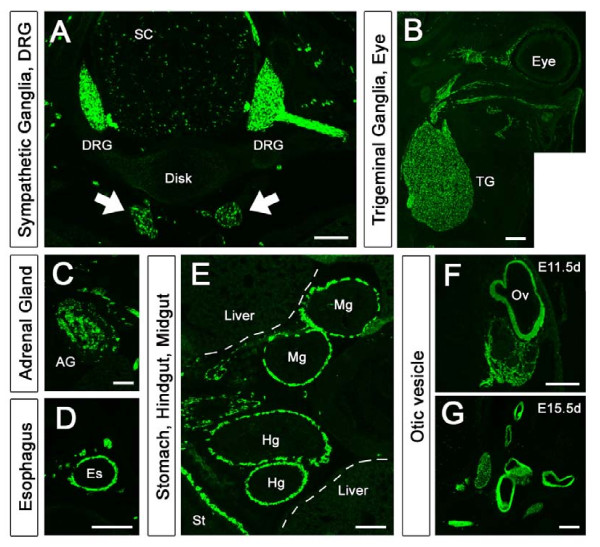
**Venus expression in neural crest lineage tissues and in otic vesicle observed without immunostaining**. The distribution of Venus^+ ^cells in *Sox10-*Venus mice was evaluated in embryonic-stage cryosections. Venus fluorescence was directly observed without immunostaining. (A) On the thoracic region of an E15.5 d axial section, Venus^+ ^cells were localized to the DRG and its central and peripheral branches. In the spinal cord, Venus^+ ^cells were broadly localized to both the gray matter and the white matter. Venus^+ ^sympathetic ganglion cells were also found at the ventral side of the vertebral body (arrows). (B) In an E15.5 d horizontal section of the cranial area, Venus^+ ^cells concentrated and formed the trigeminal ganglia (the nucleus of the V cranial nerve) and its first branch, the ocular nerve, also visualized by Venus. (C) Venus^+ ^cells were randomly localized in the E15.5 d adrenal gland. (D) NC-derived enteric ganglion cells were seen originating from the XII cranial nerve (Nervous Vargus) and migrating in an oral to anal direction in the alimentary tract. (D-E) On the lumbar level axial section, Venus^+ ^cells were captured in the esophagus, stomach, midgut, and hindgut in the stream of their migration. (F-G) The primary structure of the inner ear, designated as the otic vesicle, also expresses Venus in the embryonic period. The otic vesicle is not NC-derived, but expresses Sox10. SC, spinal cord; TG, trigeminal ganglia; AG, adrenal gland; Es, esophagus; St, stomach; Hg, hindgut; Mg, midgut; Ov, otic vesicle. Scale bars (A-C, E) 100 μm, (D, F-G) 50 μm.

To confirm the Venus^+ ^cell distribution, we prepared cryosections of *Sox10-*Venus mice from each embryonic stage and observed the Venus fluorescence directly, without any antibody staining or enhancement procedure (Figure 2). In axial sections of the thoracic region of E15.5 d mouse embryos, Venus fluorescence was intense in the DRG and its proximal and peripheral branches (Figure 2A). Venus^+ ^cells were diffusely present throughout the spinal cord. Some ganglion cells were positive for Venus (Figure 2A, arrows) in the sympathetic ganglia located at the ventral side of the vertebral disc. In horizontal cranial sections from E15.5 d *Sox10-*Venus embryos, large numbers of Venus^+ ^cells accumulated to form the trigeminal ganglia (cranial nerve V) (Figure 2B). Venus was highly expressed in the ocular nerve, which is the first branch of the trigeminal nerve. Venus^+ ^cells were randomly distributed in the E15.5 d adrenal gland, most likely due to the incomplete column structure formation at this stage (Figure 2C). In the alimentary tract, Venus^+ ^enteric ganglion cells originating from the vagal nerve (cranial nerve XII) were found migrating in a rostrocaudal direction through the esophagus, stomach, midgut, and hindgut (Figure 2D and 2E). All of these tissues are well-known NC derivatives, demonstrating that the Venus fluorescence observed in the *Sox10-*Venus mice is consistent with known NC cell localization and differentiation [1-3,5]. In the otic vesicle, which is the primary structure of the inner ear nerve, cells continuously expressed Venus throughout the embryonic period (Figure 2F and 2G). The otic vesicle is not a NC derivative, but Sox10 protein expression in otic vesicle cells during the embryonic stage has been reported [28,29].

### Cell type evaluation of the Venus^+ ^cells in *Sox10-*Venus mice

**Figure 3:**
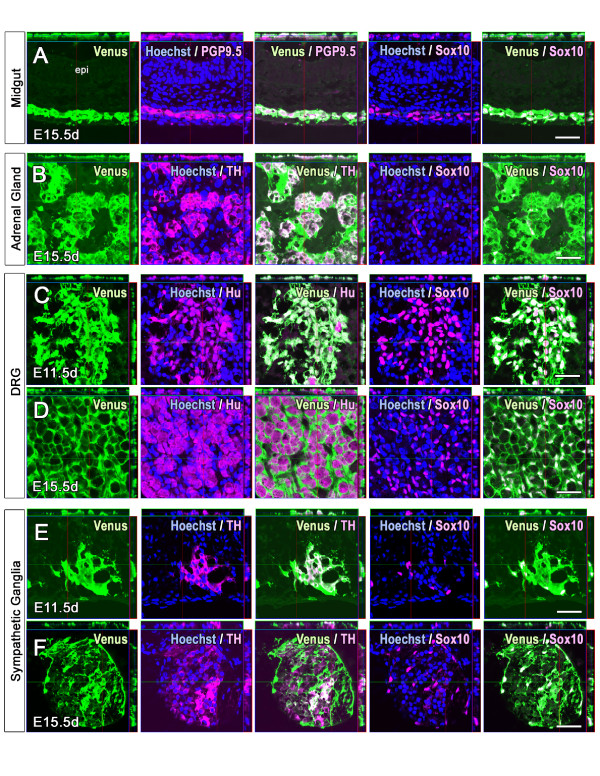
**Venus expression faithfully reflects endogenous Sox10 expression**. Immunohistochemistry was used to confirm the *in vivo *co-localization of Venus with specific cell-type markers in *Sox10-*Venus mice. (A-F) All Sox10-positive cells were also positive for Venus; no ectopic Venus expression was detected in an entire series of sections. (A) In the E15.5 d alimentary tract, PGP9.5-positive enteric ganglion cells were also positive for Venus. (B) TH-positive endocrine cells in the E15.5 d adrenal gland were invariably positive for Venus. (C-D) While all Hu-positive early embryonic DRG neurons expressed endogenous Sox10 and Venus protein at E11.5 d, mature DRG neurons lost the Sox10 and Venus expression simultaneously at E15.5 d, and Sox10^+ ^satellite cells became positive for Venus. (E-F) In the embryonic sympathetic ganglia, all the TH^+ ^neurons were also Venus^+ ^at E11.5 d, whereas TH^+^/Venus^- ^cells appeared at E15.5 d. Hu^+^, terminally differentiated neuron; PGP9.5^+^, enteric ganglion cell; TH^+^, adrenal gland endocrine cells and sympathetic ganglion neurons; epi, epithelium. Scale bars (A-F) 50 μm.

To verify that the Venus expression correctly reflects the endogenous expression of the Sox10 protein *in vivo*, we performed immunohistochemistry of E11.5 d to postnatal 1-week-old (P1 w) whole mouse frozen sections. Without exception, in all the tissues and stages examined, all the Sox10-positive cells expressed Venus. In addition to Sox10 immunostaining, we also examined cell-type-specific markers in NC-derived and Sox10^+ ^tissues. The enteric ganglion cells in the alimentary tract stained with PGP9.5 antibodies. In the esophagus, stomach, midgut, and hindgut, most of the migrated enteric ganglion cells were positive for Venus at E15.5 d (Figure 3A). In the E15.5 d adrenal gland stained for tyrosine hydroxylase (TH), a marker for catecholaminergic cells, TH-positive endocrine cells were positive for Venus (Figure 3B). In the early embryonic (E11.5 d) DRG, all Hu-positive sensory neurons were co-labeled with Sox10 and Venus (Figure 3C). These observations confirmed that *Sox10-*Venus is a useful reporter strain, in which Venus expression faithfully reflects the endogenous Sox10 *in vivo *expression.

### Loss of Venus-fluorescence correlates with the shutdown of endogenous Sox10 expression

To evaluate the on/off switching of reporter gene activity in *Sox10-*Venus mice, we verified the Sox10 expression with immunohistochemistry, and examined its correlation with Venus fluorescence. At the late embryonic stage of E15.5 d, the loss of Sox10 expression in Hu-positive DRG neurons coincided with a dramatic decrease in Venus fluorescence, compared to the early embryonic stage (Figure 3C and 3D). A similar phenomenon was observed in embryonic sympathetic ganglia: most of the TH^+ ^neurons were Venus^+ ^in the early embryonic stage, while TH^+ ^neurons in the late embryonic stage lost both Venus and Sox10 expression (Figure 3E and 3F).

The prompt on/off switching of reporter gene activity is an important characteristic of this transgenic mouse strain. In mice in which the NC lineage is labeled, such as the *Wnt1*-Cre/CAG-CAT-EGFP double transgenic mouse, NC progeny expressing Cre are indelibly labeled with the reporter gene. At the late embryonic stage of E15.5 d, neurons in the DRG and sympathetic ganglia of *Wnt1*-Cre/CAG-CAT-EGFP mice were still labeled with EGFP fluorescence even though endogenous Sox10 expression had already diminished (Additional file 4). In contrast, the sensitive on-off switching of the Venus-fluorescent reporting makes the *Sox10-*Venus mouse an accurate, real-time reporter strain.

### Identification of Venus^+ ^cells in the intact embryonic spinal cord

**Figure 4:**
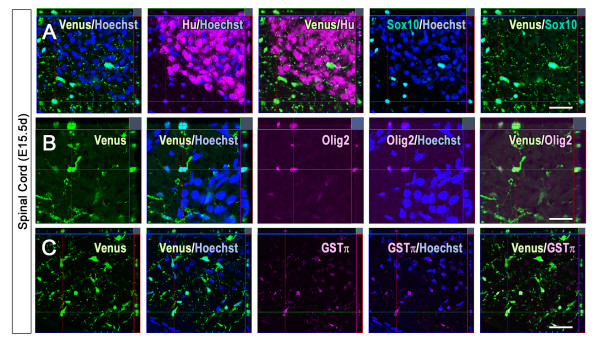
***Sox10-*Venus^+ ^cells in the intact embryonic spinal cord**. Immunohistochemical analysis of Venus^+ ^cells in the intact embryonic spinal cord of *Sox10-*Venus mice. (A) Venus fluorescence did not colocalize with the pan-neuronal marker Hu in the ventral area of *Sox10-*Venus mice. (B-C) These Venus^+ ^cells expressed the oligodendroglial lineage markers GSTπ and Olig2, showing that the Venus^+ ^cells were OPCs and mature oligodendrocytes. Scale bars; (A-C) 50 μm.

Previous reports have suggested that Sox10 is expressed in oligodendrocyte progenitor cells (OPCs) and mature oligodendrocytes [24-26]. We examined the identity of the Venus^+ ^cells observed in the ventral region of the intact embryonic spinal cord of *Sox10-*Venus mice by immunohistochemistry. The Venus^+ ^cells were negative for the pan-neuronal marker Hu (Figure 4A), but positive for the oligodendroglial-lineage markers GSTπ and Olig2 (Figure 4B and 4C). Therefore, in the spinal cord of the embryonic *Sox10-*Venus mouse, Venus labels oligodendroglial-lineage cells without distinction as to their degree of maturation.

### Venus^+ ^cells in the intact and injured spinal cords of adult *Sox10-*Venus mice

**Figure 5:**
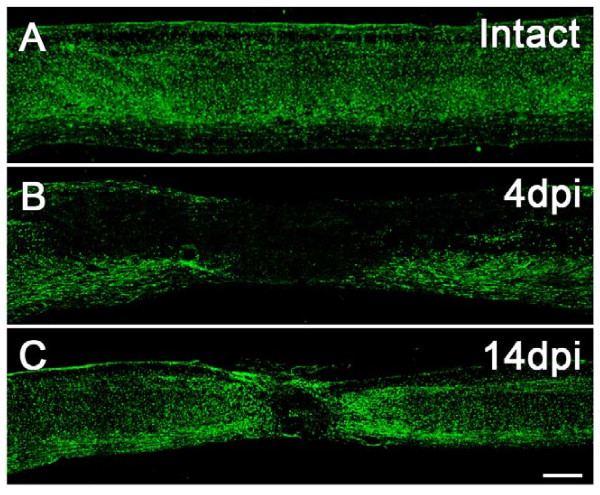
***Sox10-*Venus^+ ^cells actively accumulate at the spinal cord injury site**. Venus fluorescence illuminated the behavior of *Sox10- *expressing cells after a spinal cord injury. (A) In an intact adult mouse spinal cord, Venus^+ ^cells were clearly visible in both in the gray and the white matter. (B) In the acute phase of contusive spinal cord injury (4 dpi; days-post-injury), Venus^+ ^cells were absent from the lesion site. (C) In the subacute phase (14 dpi), numerous Venus^+ ^cells were present around the lesion and delineated the lesion epicenter. Scale bars (A-C) 500 μm.

To examine the phenotype of Venus^+ ^cells in intact and injured spinal cords of *Sox10-*Venus mice, we first observed changes in the Venus^+ ^cells over time (Figure 5A - C). After spinal cord injury (SCI), there was a sharp drop in the number of Venus^+ ^cells, followed by a gradual accumulation around the lesion epicenter at later stages after the injury.

**Figure 6:**
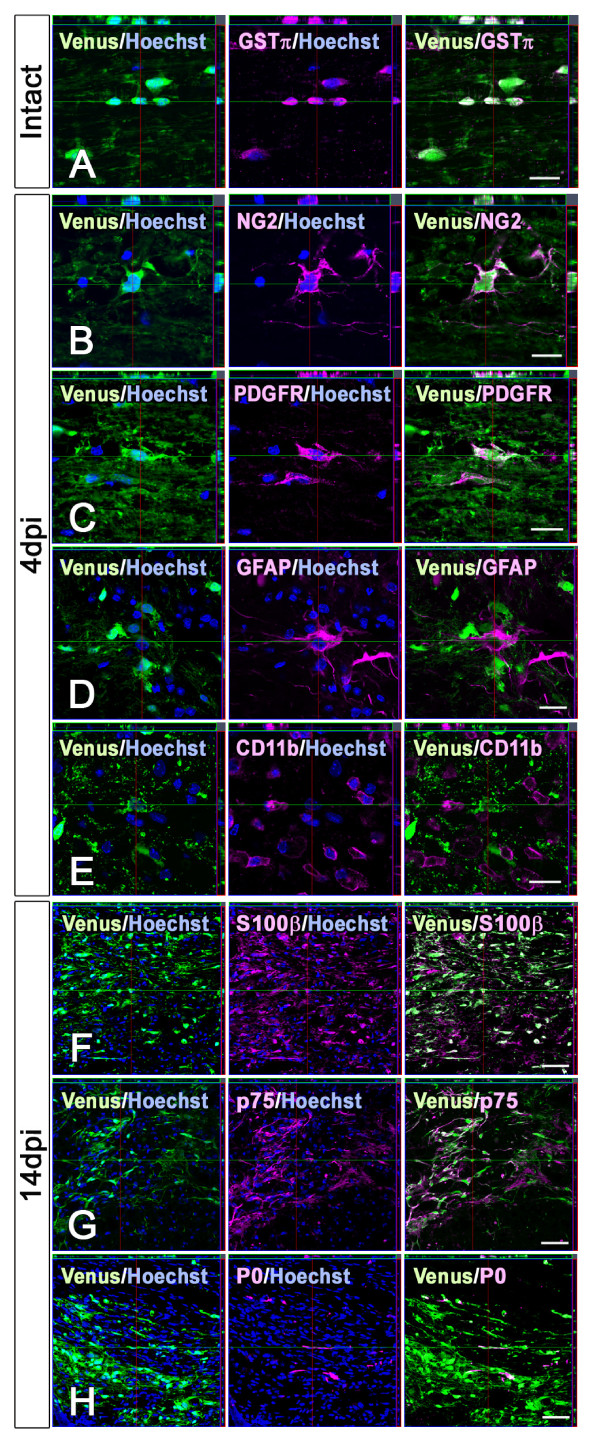
**Immunohistochemical analysis of *Sox10-*Venus^+ ^cells after spinal cord injury**. (A-E) OPCs and mature oligodendrocytes were Venus^+ ^in the intact and injured spinal cord of *Sox10-*Venus mice. (A) Most Venus^+ ^cells were positive for GSTπ in the intact spinal cord. (B) In the acute phase (4 dpi), NG2^+ ^cells increased in the residual white matter in response to the SCI. Some of the NG2^+ ^cells also expressed Venus. (C-E) In the acute phase, Venus^+ ^oligodendroglial cells were also positive for PDGFRα (C), but were negative for GFAP (D) and CD11b (E). (F) In the subacute phase of the SCI (14 dpi), S100β^+ ^cells were also present around the lesion site, and expressed Venus. (G) Some of the Venus^+ ^cells were positive for p75, suggesting that they were immature Schwann cells with bipolar processes. (H) A small number of Venus^+ ^cells were mature Schwann cells, as assessed by Protein Zero expression. Scale bars; (A-E) 20 μm, (F-H) 50 μm.

Next, we identified the Venus^+ ^cells in intact and injured spinal cords by immunostaining with various cell markers (Figure 6A - H). In the intact spinal cord, Venus^+ ^cells were easily visualized in both the gray and white matter (Figure 5A), and were mostly positive for GSTπ (Figure 6A). After contusive SCI, Sox10-expressing cells disappeared from the lesion epicenter (Figure 5B). In the acute phase of SCI (4 days-post-injury; 4 dpi), cells positive for NG2 and Venus appeared in the residual white matter (Figure 6B). Although NG2 is also known to stain reactive astrocytes and macrophages [30], the Venus^+ ^cells observed in the injured spinal cord were negative for GFAP (Figure 6D) and CD11b (Figure 6E), indicating that they were Sox10-expressing oligodendrocytes. We also observed Venus^+ ^cells that were positive for PDGFRα, an OPC marker (Figure 6C), suggesting that OPCs also expressed Sox10 in the injured spinal cord.

In the later subacute phase of SCI (14 dpi), numerous Venus^+ ^cells were found in the epicenter and around the lesion site (Figure 5C). Immunohistochemistry revealed that many of the Venus^+ ^cells were positive for S100β, a marker of the Schwann cell lineage, confirming Schwann cell involvement after SCI (Figure 6F). Some of the Venus^+ ^cells had bipolar processes and were positive for p75, suggesting that they were immature Schwann cells (Figure 6G), while a few Venus^+ ^mature Schwann cells were also identified by their P0 expression (Figure 6H). The observation that both immature and mature Schwann cells were Venus^+ ^in the injured spinal cord is consistent with the Schwann cell lineage deriving from the NC.

### Comparison of the *Sox10-*Venus strain with other NC reporter mouse strains

The data from several NC reporter mouse lines, such as *Sox10*(S4F)-Cre, *P0*-Cre, *Wnt1*-Cre, and *Ht-Pa*-Cre [5,14,16,26,31,32], have already been published. To compare this body of data to the observations gained from the *Sox10-*Venus mouse, the embryonic expression patterns of various NC-derived tissues are summarized for each strain in Table 1. At the early embryonic period (E11.5 d - E12.5 d), the reporter gene activity of all the mouse strains was quite similar. All the strains demonstrated reporter gene activity in numerous NC-lineage tissues, including the DRG, sympathetic ganglia, melanoblasts, enteric nervous system, superior/jugular ganglion, aorta, and craniofacial mesenchyme. All the strains also showed reporter expression in the otic vesicle, which does not originate from the NC.

**Table 1:** Comparison of reporter expression in *Sox10-*Venus and various other mice with NC lineage-labeling

Tissues	*Sox10*-Venus	*Sox10*-Cre	*P0*-Cre	*Wnt1*-Cre	*Ht-Pa-*Cre
Dorsal root ganglia	+	+	+	+	+
Sympathetic ganglia	+	+	+	+	+
Melanoblasts	+	+	+	+	+
Enteric nervous system	+	+	+	+	+
Superior/jugular ganglion	+	+	+	+	+
Aortae	+	+	+	+	+
Craniofacial mesenchyme	+	+	+	+	+
Otic vessicle	+	+	+	+	+
Oligodendroglia	+	+	-	-	-
Ventral neural tube	+	+	-	-	-
Developing limb	+	+	-	-	-

Reporter gene expression patterns at the embryonic stage are compared in various mice developed to trace neural crest derivatives: *Sox10-*Venus, *Sox10*(S4F)-Cre [26], *P0*-Cre [5,15], *Wnt1*-Cre [14,16,26,31,32], and *Ht-Pa-*Cre [16,26]. All these lines have similar labeling patterns in both NC-lineage tissues (DRG, sympathetic ganglia, melanoblasts, enteric nervous system, superior/jugular ganglion, aorta, craniofacial mesenchyme) and in tissues not in the NC lineage (otic vesicle, oligodendroglial cells, and OPCs). In the developing limbs of *Sox10-*Venus and *Sox10-*Cre mice, which reflect the endogenous Sox10 expression profile, the reporter fluorescence clearly labeled oligodendroglial cells and the peripheral nervous network. Immunohistochemistry did not detect any ectopic reporter gene expression in any tissues from the embryonic *Sox10-*Venus mice.

Regarding the differences in these reporter strains, CNS myelinating glial cells were not labeled in the *P0*-Cre, *Wnt1*-Cre, and *Ht-Pa*-Cre strains; they were only labeled in the *Sox10*-related strains *Sox10-*Venus and *Sox10*(S4F)-Cre. In the brain and spinal cord, the labeling of oligodendroglial cells and OPCs in the *Sox10-*Venus and *Sox10-*Cre mice reflected the endogenous Sox10 expression in these cells, since they are not derived from the NC. The peripheral nerve network in developing limbs consists of nerve fibers and their associated Schwann cells, which are both derived from the NC. Although the *LacZ *expression in the developing limb was ambiguous in the *Sox10*(S4F)-Cre/Rosa-*LacZ *mouse [26], the Venus fluorescence observed in the same region with the *Sox10-*Venus mouse clearly originated from the Schwann cells (Figure 1E).

## Discussion

In this study, we developed and characterized the *Sox10-*Venus BAC transgenic mouse. Analysis of the early embryonic stages showed that not only NC lineage cells, but also oligodendroglial cells were clearly labeled with high-intensity Venus fluorescence (Figure 1 and 2). Compared to other published transgenic and knock-in reporter mouse strains, the *Sox10-*Venus mouse has advantages that make it invaluable for future studies.

The mice with NC-lineage tracing *Wnt1*-Cre, *P0*-Cre, and *Ht-PA*-Cre, crossed with those with Cre/loxP-mediated cell labeling, Rosa-*LacZ *and CAG-CAT-EGFP, can be used only for analyzing cell lineage or migration, because the target cells are irreversibly labeled [5,14-20]. Although *Sox10-*Venus also labels NC-lineage cells, the fast on/off switching of Venus fluorescence is tightly correlated with endogenous Sox10 expression. Similarly, the reporter gene activity in *CNP-*EGFP and *PLP-*EGFP mice, which are transgenic mice that label oligodendroglial-lineage cells, truly reflects the *in vivo *expression of specific genes driven by specific promoters or enhancers. The transgenic system of the *Sox10-*Venus mouse is quite similar to these; however, the Venus fluorescence is brighter and more intense than EGFP fluorescence.

Although reporter gene activity occurs in NC cells and oligodendrocytes in all *Sox10 *reporter mouse lines, i.e., *Sox10^LacZ/+^*, *Sox10-*rtTA, *Sox10-*Cre strains [24-26], and the new *Sox10-*Venus strain, each line has advantages and disadvantages. *LacZ *knock-in *Sox10*^*LacZ*/+ ^heterozygous pups are prone to spontaneous mutation phenotypes due to haploinsufficiency. Also, to observing the *LacZ *expression in *Sox10*^*LacZ*/+ ^mice requires additional visualization procedures, making live cell imaging difficult. The *Sox10-*rtTA knock-in crossed with the inducible TRE-*LacZ *transgenic is unique, but the reporter expression is transient and does not fluoresce, making it difficult to observe directly. The *Sox10-*Cre/CAG-CAT-EGFP double transgenic mouse traces both NC and oligodendrocyte progeny, since it reports past as well as ongoing Sox10 expression. With this double transgenic mouse, it is possible to carry out cell sorting or live imaging.

The *Sox10-*Venus strain overcomes most of the disadvantages of the above-mentioned *Sox10 *reporter mouse lines. In this mouse, the intense Venus fluorescence can be directly observed from outside the embryo, without staining or enhancement procedures (Figure 1, 2, and movies in Additional Files 2 and 3). Venus expression faithfully reflects real-time endogenous Sox10 expression, with prompt on/off switching (Figure 3). Although the choice of strain obviously depends on the purpose of the study, we believe that the *Sox10-*Venus mouse is the most appropriate reporter line for numerous fields of research.

Although *in vivo *time-lapse imaging of oligodendroglial cell migration (including OPCs) has recently been reported, it has only been conducted in zebrafish [33,34]. Accurate time-lapse imaging has been difficult to conduct in mice until now. There are several issues that need to be considered in such studies. For instance, (1) Is the *normal *and *natural *behavior of the cells or tissues of interest being observed? In some cases, the invasive procedures required to complete the imaging, including surgical incision, tissue-slice culture, electroporation, dye injection, and virus infection, may affect the tissues observed. (2) To what degree does the transgenic manipulation affect the phenotype? In *Sox10*^*LacZ*/+ ^mice, the resulting phenotype, coupled with haploinsufficiency, complicates analysis of the area of interest. Our analysis of the *Sox10-*Venus transgenic line demonstrates that it has neither developmental defects nor ectopic Venus expression. Thus, the *Sox10-*Venus strain appears to be a useful tool for investigating normal developmental processes via live cell monitoring, using directly observed fluorescence without invasive intervention procedures (Figure 1G, movies in Additional Files 2 and 3).

Sox10 is a well-known marker of neural crest stem cells (NCSCs), along with *slug, snail*, and *p75 *[35,36]. There have been numerous reports recently of NCSCs surviving in a wide range of tissues through the entire lifespan of the animal, suggesting that NCSCs may have the potential to support the regeneration and recovery of damaged tissues. The Venus fluorescence in *Sox10-*Venus mice will make it possible to prospectively sort Venus^+ ^cells by flow-cytometry and collect an enriched population of NCSCs. Since NCSCs are located in easily accessible peripheral tissues such as the skin and bone marrow, NCSCs have been receiving increasing attention for future clinical applications in cell transplantation therapy, because the feasibility of autologous transplantation is anticipated [37-39]. Autologous cell transplantation therapy avoids the immunological and ethical concerns related to the use of embryonic stem cells. We hope that the *Sox10-*Venus strain will prove to be a powerful tool for enhancing the progress of NCSC research.

The mouse is an excellent model system for studying human disease progression and pathogenesis. We demonstrated the usefulness of our new reporter mouse strain *Sox10-*Venus for monitoring processes occurring after a traumatic disorder (Figure 5 and 6), and crossing it with mutant mouse lines may provide insight into the processes behind numerous developmental defects. As with the analysis conducted in CNP-EGFP mice to study the behavior of oligodendroglial cells in SCI [30], *Sox10-*Venus mice have potential applications not only for oligodendrocyte research, but also for all Sox10^+^-tissue analyses, including disorders of the NC cells and peripheral nerves. The ability to visualize the processes of disease initiation and progression will help to shed light on the pathophysiology of many human diseases.

## Conclusions

We developed the novel *Sox10-*Venus BAC transgenic mouse line, in which Venus fluorescence labels NC and oligodendroglial lineage cells. Endogenous Sox10 expression is accurately reported by Venus fluorescence over the course of normal development, without ectopic Venus expression. The highly intense reporter fluorescence allows for *in vivo *imaging of single-cell migration. This strain will be especially useful for analyzing spinal cord injury and studying NCSCs.

## Methods

### BAC Construction and development of *Sox10-*venus BAC transgenic mice

For generating the *Sox10*-Venus transgenic mouse, we prepared the following BAC construction. Detailed procedures will be described elsewhere (CA, TI, YUI, manuscript in preparation). Briefly, a BAC clone *RP24-85O14 *(CHORI, BACPAC Resources) that covers 225.6 kb territory of mouse *Sox10 *gene locus was electroporated into the recombinogenic bacterial strain EL250 for systematic modifications [40]. To obtain in-frame replacement of the exon containing the *Sox10 *translation initiation codon (ATG) to the Venus-polyadenylation signal (pA) cassette in the BAC clone, 1.2 kb homology arms were amplified by PCR and sequentially subcloned into the pBluescriptII vector (Stratagene). Then the Venus-pA-FRT-*Kanamycin*-resistant gene (*Kan^r^*)-FRT cassette was inserted in between the arms and the fragment containing the Venus-pA-*Kan^r ^*cassette with the homology arms was isolated by agarose gel electrophoresis for homologous recombination which was processed in EL250 as was reported previously [41]. Precise insertion of the Venus-pA cassette and excision of the *Kan^r ^*in the *Sox10*-BAC was verified by PCR as well as pulse field gel electrophoresis and the modified BAC clone was linearlized by PI-*Sce*I, dialyzed and diluted to ~2 ng/μl for pronuclear injections as previously described [41]. For BAC transgenic mouse founders, presence of *RP24 *BAC vector sequences immediately upstream or downstream of the PI-*Sce*I site was always examined by PCR tail DNA analyses to minimize the possibility that fortuitous deletions occur on the BAC transgene after the chromosomal integrations [41]. The established BAC transgenic mouse lines were bred with wild-type C57BL/6J mice and the tail DNA was used for the PCR genotyping: The PCR primers for the transgene detections are provided in Table S1; Additional File 5.

### Animals

We purchased wild-type C57BL/6J and ICR mice from SLC Japan. The Cre-expressing transgenic lines, *Wnt1*-Cre [14] and *P0*-Cre [15], were mated with an EGFP reporter line (CAG-CAT-EGFP [18]) to obtain *Wnt1*-Cre/CAG-CAT-EGFP and *P0*-Cre/CAG-CAT-EGFP double-transgenic mice. All mice were housed under specific pathogen-free conditions. All experimental procedures were approved by the Institutional Animal Care and Use Committee of Keio University, Murayama Medical Center, and Tokyo Medical and Dental University. All surgical interventions and animal care procedures were in accordance with the Laboratory Animal Welfare Act, the Guide for the Care and Use of Laboratory Animals (National Institute of Health, USA).

### Histological analysis

Immunohistochemical analyses were performed as described previously [5,42,43]. Briefly, E9.5 d, n = 3; E10.5 d, n = 10; E11.5 d, n = 9; E12.5 d, n = 5; E13.5 d, n = 6; E14.5 d, n = 3; E15.5 d, n = 7; E16.5 d, n = 3; P0 d, n = 3; and P1 w, n = 3 transgenic embryos (E0 = day of plug) and pups were fixed with 4% PFA, and 16-μm-thick cryosections were prepared with the cryostat CM3000 (Leica). The detailed information about the primary and secondary antibodies was described in Table S1; Additional File 5. Antigen retrieval was applied for specific targets (anti-Olig2, anti-hSox10) by incubating them with Target Retrieval Solution (Dako) in an autoclave at 105°C for 5 minutes. Fluorescence immunostaining with specific primary antibodies was performed overnight at 4°C, followed by one-hour incubation at room temperature with the appropriate secondary antibodies conjugated with Alexa488, Alexa555, or Alexa647 (Invitrogen) along with Hoechst 33258 (10 μg/ml, Sigma) for nuclear staining. The samples were examined with a laser scanning confocal microscope (LSM700 or Pascal: Carl Zeiss) or fluorescent microscope (BZ-9000: Keyence, MVX-CSU: Olympus).

### Time-Lapse Imaging

Time-lapse observation (Figure 1G, movies in Additional Files 2 and 3) was performed as described previously [44,45]. Briefly, freshly isolated embryos were collected in cold phosphate buffered saline (PBS) and genotyped based on their Venus fluorescence, using an inverted epifluorecence microscope (Leica MZ10F). The embryos were transferred with 100 - 150 µl of enriched medium to the center of a 35-mm glass-bottom dish (hole size 27 mm; Matsunami) and mixed with 100-150 µl of type I collagen-gel solution (Cellmatrix IA; Nitta Gelatin). The solution had been previously diluted to 0.5-0.6 mg/ml with distilled water, 5× DMEM, and a neutralizing buffer, with a final concentration about 0.3 mg/ml, according to the manufacturer's protocol. The gel was incubated at 37°C for 20 minutes. Once the gel was solidified, up to 600 μl of imaging culture medium was gently added to the gel. The final concentration of the culture medium was as follows: DMEM/F-12 (1:1), glucose (0.6%), L-glutamine (2 mM), sodium bicarbonate (3 mM), HEPES buffer (50 mM), insulin (25 μg/ml), transferrin (100 μg/ml), progesterone (20 nM), sodium selenate (30 nM), and putrescine (60 μM), with equilibration buffer supplemented with 4% fetal bovine serum. The dish was attached to a pre-heated microscope stage and incubated at 37°C in 5% CO_2_. Imaging was carried out with an epifluorescence microscope from Keyence (BZ9000) or a confocal Carl Zeiss microscope (LSM5 PASCAL Exciter). Images were acquired with a 10× objective lens every 10 or 20 minutes, and processed using the Keyence Bz-II application and Zeiss LSM5 software, respectively. NIH ImageJ 1.44e and Quicktime Pro 7.6.8 were used for preparing movie files.

### Spinal cord injury model

Adult female *Sox10-*Venus mice (8-weeks old, n = 4 for each time point) were anesthetized with an intraperitoneal injection of ketamine (100 mg/kg) and xylazine (10 mg/kg). The dorsal surface of the dura mater was exposed after laminectomy at the tenth thoracic vertebra, and SCI was induced using an IH (Infinite Horizon) impactor (60 kDyn; Precision Systems & Instrumentation) as described previously [46,47]. The mice were returned to their home cages with free access to water and food. Intact mice and mice at 4 and 14 days post-injury (dpi) were deeply anesthetized and transcardially perfused with chilled PBS followed by 4% paraformaldehyde (PFA). The spinal cords were removed and immersed in 4% PFA at 4°C overnight, and then immersed in 15% and 30% sucrose for 24 hours each, at 4°C. The spinal cords were then embedded in OCT compound and sectioned into 12-µm-thick sagittal sections by cryostat (Leica CM3000).

## Abbreviations

NC: neural crest; OPC: oligodendrocyte progenitor cell; HMG: high mobility group; PNS: peripheral nervous system; CNS: central nervous system; P0: protein zero; EGFP: enhanced green fluorescent protein; CNP: 2'-3'-cyclic nucleotide 3'-phosphodiesterase; PLP: myelin proteolipid protein; TH: tyrosine hydroxylase; DRG: dorsal root ganglia; SCI: spinal cord injury; DPI: days-post-injury; p75: p75 neurotrophin receptor; NSPC: neural stem/progenitor cell; NCSCs: neural crest stem cells; PBS: phosphate buffered saline.

## Competing interests

The authors declare that they have no competing interests.

## Authors' contributions

SS, CA, and HO designed the project. SS, AY, FRM, SS, TI, YUI, NN, and MS performed experiments and analyzed the data. SS, AY, and FRM prepared the figures. SS, AY, FRM, HK, TI, MN, CA, and HO wrote the manuscript. MN, CA, and HO supervised the project. All authors read and approved the final manuscript.

## Supplementary Material

Additional file 1 **Embryonic age-dependent fluorescence changes compared among the *Sox10-*Venus, P0-Cre/CAG-CAT-EGFP, and *Wnt1*-Cre/CAG-CAT-EGFP mouse strain**. (A-C) Venus fluorescence changes over time were observed from outside of the *Sox10-*Venus embryo. Deep-tissue fluorescence gradually decreased from E11.5 d to E15.5 d (see Figure 1 for additional photos). (C-E) At E14.5 d, reporter gene expression patterns were quite similar between the transgenic *Sox10-*Venus mouse and the double-transgenic mice *P0*-Cre/CAG-CAT-EGFP and *Wnt1*-Cre/CAG-CAT-EGFP. Scale bars (A-E) 1.0 mm. Click here for file

Additional file 2 **Live imaging of single-cell movement: *Sox10-*Venus^+ ^NC-derivatives in the superficial embryonic skin layer (1)**. Time-lapse imaging with an epifluorescence microscope (Keyence) showed Venus-positive cell movements in E14.5 d *Sox10-*Venus embryo ex-vivo skin explants. One frame was captured every 10 minutes over a 6-hour period (motion: 6 fps). Click here for file

Additional file 3 **Live imaging of single-cell movement: *Sox10-*Venus^+ ^neural crest derivatives in the superficial embryonic skin layer (2)**. Confocal time-lapse imaging of the front facial area of the whole E10.5 d *Sox10-*Venus embryo captured isolated Venus-positive cells. The distinctive, intense Venus fluorescence made it possible to follow migrating cells within the embryo over several hours, even capturing the dynamic changes in cell shape. One frame was captured every 20 minutes using a confocal Pascal microscope (Carl Zeiss). Scale bar: 50 µm, motion: 4 fps. Click here for file

Additional file 4 **NC progeny were permanently EGFP-labeled in the *Wnt1*-Cre/CAG-CAT-EGFP mouse**. (A-B) Immunohistochemical analysis with anti-GFP and marker antibodies for specific cell types showed that the EGFP reporter gene is continuously expressed in NC derivatives in the DRG (A) and sympathetic ganglia (B) of E15.5 d *Wnt1*-Cre/CAG-CAT-EGFP mice. Scale bars (A-B) 50 μm. Click here for file

Additional file 5 Table S1: Detailed information about sequences and antibodies. Click here for file
