# Supplementary figures and images for: Sox10-Venus mice: a new tool for real-time labeling of neural crest lineage cells and oligodendrocytes
Source: Mol Brain. 2010 Oct 31;3:31. doi: 10.1186/1756-6606-3-31 (PMC2989948; doi:10.1186/1756-6606-3-31)

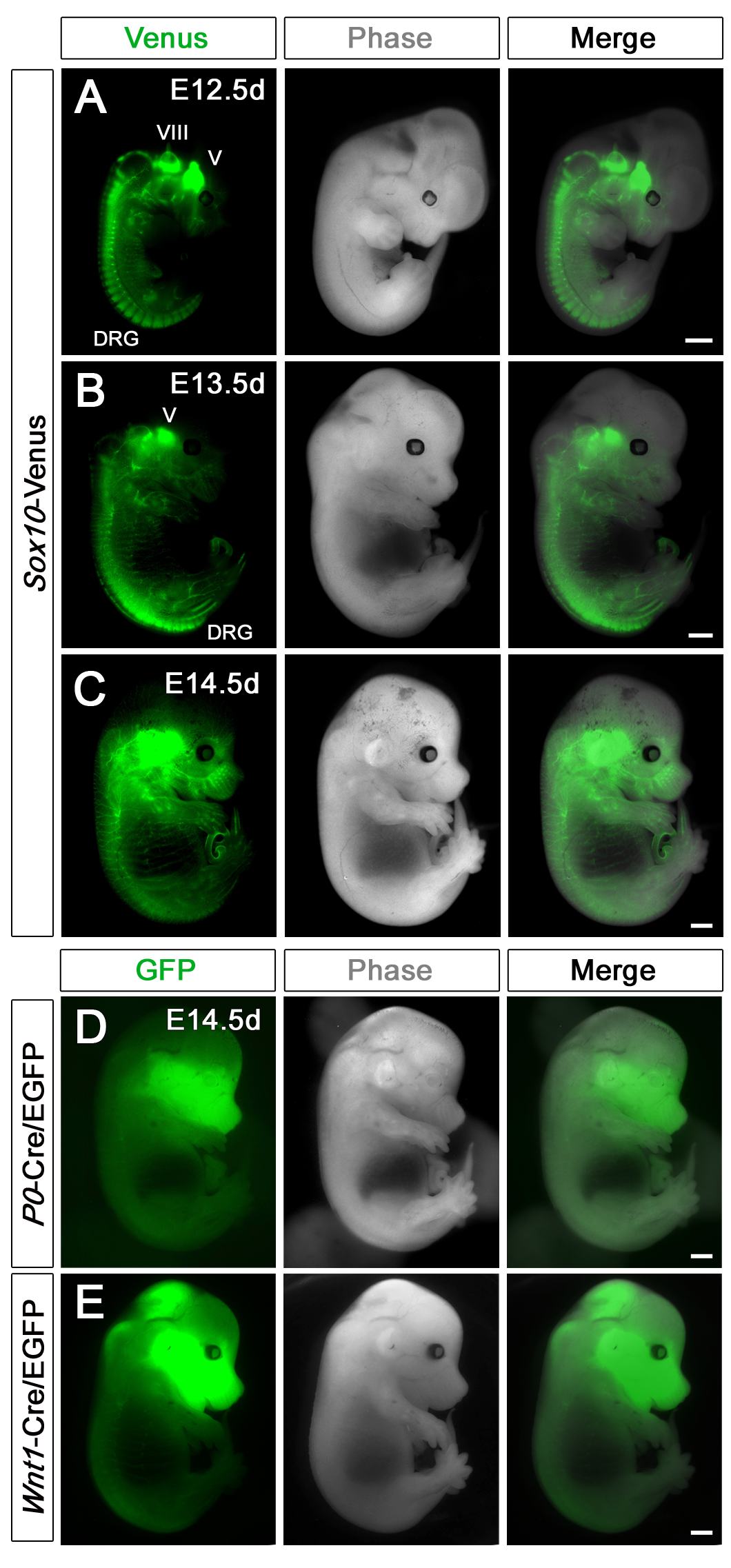

Supplement: Additional file 1 — Embryonic age-dependent fluorescence changes compared among the Sox10-Venus, P0-Cre/CAG-CAT-EGFP, and Wnt1-Cre/CAG-CAT-EGFP mouse strain. (A-C) Venus fluorescence changes over time were observed from outside of the Sox10-Venus embryo. Deep-tissue fluorescence gradually decreased from E11.5 d to E15.5 d (see Figure 1 for additional photos). (C-E) At E14.5 d, reporter gene expression patterns were quite similar between the transgenic Sox10-Venus mouse and the double-transgenic mice P0-Cre/CAG-CAT-EGFP and Wnt1-Cre/CAG-CAT-EGFP. Scale bars (A-E) 1.0 mm. [file 1756-6606-3-31-S1.TIFF]

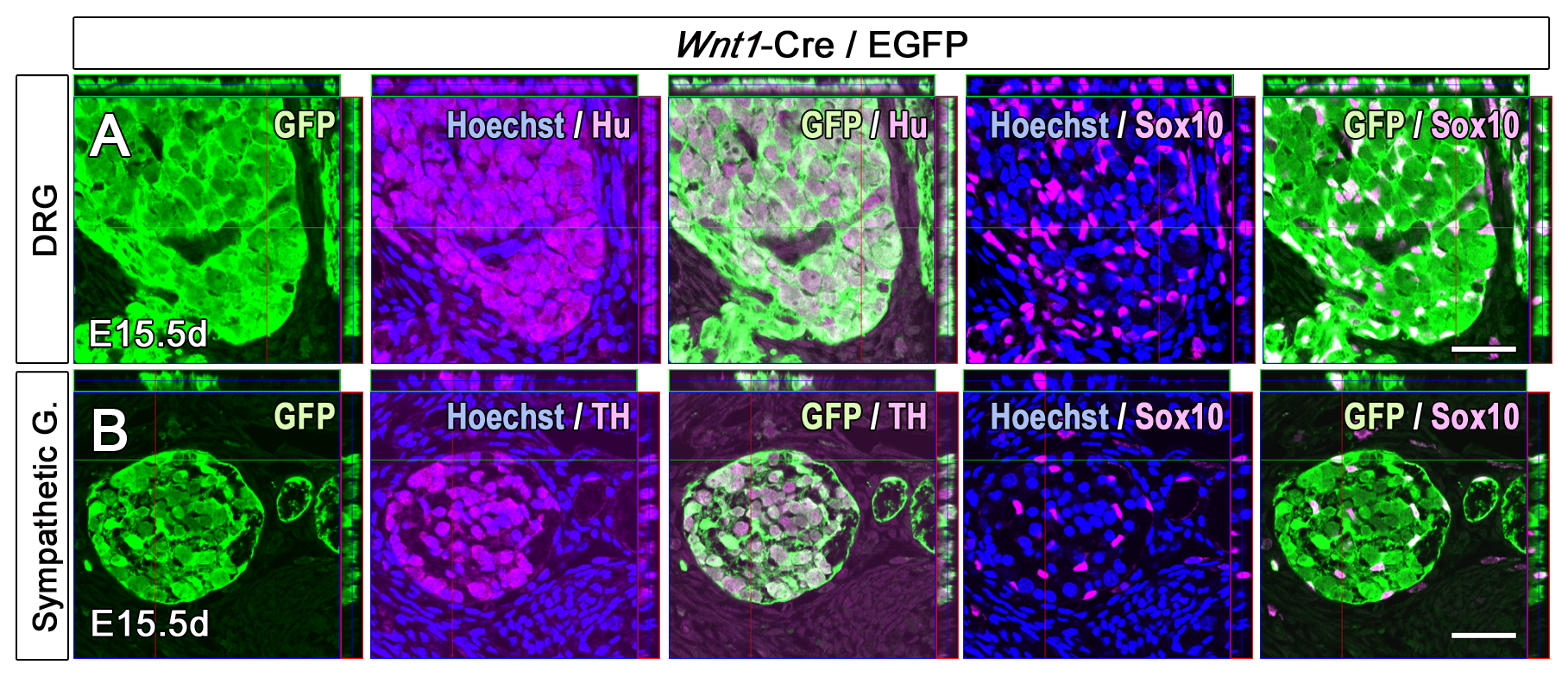

Supplement: Additional file 4 — NC progeny were permanently EGFP-labeled in the Wnt1-Cre/CAG-CAT-EGFP mouse. (A-B) Immunohistochemical analysis with anti-GFP and marker antibodies for specific cell types showed that the EGFP reporter gene is continuously expressed in NC derivatives in the DRG (A) and sympathetic ganglia (B) of E15.5 d Wnt1-Cre/CAG-CAT-EGFP mice. Scale bars (A-B) 50 μm. [file 1756-6606-3-31-S4.TIFF]
