# Supplementary material for: Sox10-Venus mice: a new tool for real-time labeling of neural crest lineage cells and oligodendrocytes
Source: Mol Brain. 2010 Oct 31;3:31. doi: 10.1186/1756-6606-3-31 (PMC2989948; doi:10.1186/1756-6606-3-31)
Supplement: Additional file 5 — Table S1: Detailed information about sequences and antibodies. [file 1756-6606-3-31-S5.DOC]

**Primer Sequence for Genotyp**ing

| **Name and Size of Primers** | **Sequence** | **PCR Product** |
| --- | --- | --- |
| SOX10-Forward (20mer) | 5’- AAGACGTGGAGGCGGGACGC -3’ | 278 bp |
| VENUS-Reverse (22mer) | 5’- CACCACCCCGGTGAACAGCTCC -3’ |

**Antibodies** for Immunostaining

| **Antibodies (Conjugation)** | **Dilution** | **Origin (Company)** |
| --- | --- | --- |
| Goat polyclonal anti-hSOX10 | IHC(1:200) | R&D |
| Rabbit polyclonal PGP9.5 | IHC(1:2000) | Cosmo Bio |
| Human polyclonal anti-HU | IHC(1:2000) | Gift from Dr. Robert Darnell |
| Rabbit polyclonal anti-TH | IHC(1:100) | Chemicon |
| Goat polyclonal anti-Olig2 | IHC(1:200) | R&D |
| Mouse monoclonal anti-GSTπ | IHC(1:200) | BD bioscience |
| Rabbit polyclonal anti-NG2 | IHC(1:200) | Chemicon |
| Rabbit polyclonal anti-PDGFRα | IHC(1:200) | Santa Cruz |
| Rat monoclonal anti-GFAP | IHC(1:200) | Invitrogen |
| Rat monoclonal anti-CD11b | IHC(1:200) | Serotec |
| Mouse monoclonal anti-S100β | IHC(1:500) | Sigma |
| Rabbit polyclonal anti-p75 | IHC(1:500) | Chemicon |
| Chick polyclonal anti-P0 | IHC(1:500) | AVES Labs |
| Rabbit polyclonal anti-GFP | IHC(1:500) | MBL |
| Goat polyclonal anti-GFP | IHC(1:200) | Rockland |
| Chick polyclonal anti-GFP | IHC(1:500) | AVES Labs |
| Donkey anti-Goat IgG (Alexa488/555/647) | IHC(1:800) | Molecular Probes |
| Donkey anti-Mouse IgG (Alexa488/555) | IHC(1:800) | Molecular Probes |
| Donkey anti-Rabbit IgG (Alexa488/555/647) | IHC(1:800) | Molecular Probes |
| Goat anti-Chick IgG (Alexa488) | IHC(1:800) | Molecular Probes |
| Goat anti-Human IgG (Alexa488/647) | IHC(1:800) | Molecular Probes |
